# Immunohistochemical Loss of the DNA Mismatch Repair Proteins MSH2 and MSH6 in Malignant Fibrous Histiocytomas

**DOI:** 10.1155/2004/735237

**Published:** 2004-12-22

**Authors:** Kajsa Ericson, Jacob Engellau, Annette Persson, Annika Lindblom, Henryk Domanski, Måns Åkerman, Mef Nilbert

**Affiliations:** ^1^ Department of Oncology, the Jubileum Institution, University Hospital, Lund SE-221 85, Sweden; ^2^ Department of Pathology, University Hospital, Lund, Sweden; ^3^ Department of Clinical Genetics, Karolinska Hospital, Stockholm, Sweden

## Abstract

*Purpose*: Soft tissue sarcomas (STS) account for less than 1% of all malignancies and constitute a heterogeneous tumor entity in which malignant fibrous histiocytomas (MFH) represent one-third and are characterized by a lack of type-specific differentiation. A defective mismatch repair (MMR) system cause the familial cancer syndrome hereditary non-polyposis colorectal cancer (HNPCC), and since occasional MFH have been described in HNPCC patients we assessed the contribution of defective MMR to the development of MFH.

*Methods*: MMR status was characterized in a series of 209 histopathologically reviewed MFH. Tissue microarray sections from the tumors were immunohistochemically stained for the MMR proteins MLH1, MSH2 and MSH6, and cases with aberrant staining were further characterized for microsatellite instability.

*Results and Discussion*: Two of the 209 STS–a storiform-pleomorphic MFH and a myxofibrosarcoma–showed concomitant loss of MSH2 and MSH6, but retained staining for MLH1 on both cases. The myxoid tumor also had a microsatellite unstable phenotype. These findings, together with previous observations of defective MMR in pleomorphic STS, indicate that these tumors may be part of the HNPCC-associated tumor spectrum and demonstrate that MMR defects occur in a small subset of STS.


*Sarcoma*, December 2004, *VOL. 8, NO. 4, 123–127*

Taylor & Francis healthsciences

ORIGINAL ARTICLE

### Immunohistochemical loss of the DNA mismatch repair proteins MSH2 and MSH6 in malignant fibrous histiocytomas

KAJSA ERICSON[1], JACOB ENGELLAU[1], ANNETTE PERSSON[2], ANNIKA LINDBLOM[3], HENRYK DOMANSKI[2], MÅNS ÅKERMAN[2] & MEF NILBERT[1]

*Departments of [1]Oncology and [2]Pathology, University Hospital, Lund, and [3]Department of Clinical Genetics, Karolinska Hospital, Stockholm, Sweden*

**Abstract**

*Purpose:* Soft tissue sarcomas (STS) account for less than 1% of all malignancies and constitute a heterogeneous tumor entity in which malignant fibrous histiocytomas (MFH) represent one-third and are characterized by a lack of type-specific differentiation. A defective mismatch repair (MMR) system cause the familial cancer syndrome hereditary non-polyposis colorectal cancer (HNPCC), and since occasional MFH have been described in HNPCC patients we assessed the contribution of defective MMR to the development of MFH.

*Methods:* MMR status was characterized in a series of 209 histopathologically reviewed MFH. Tissue microarray sections from the tumors were immunohistochemically stained for the MMR proteins MLH1, MSH2 and MSH6, and cases with aberrant staining were further characterized for microsatellite instability.

*Results and Discussion:* Two of the 209 STS – a storiform-pleomorphic MFH and a myxofibrosarcoma – showed concomitant loss of MSH2 and MSH6, but retained staining for MLH1 on both cases. The myxoid tumor also had a microsatellite unstable phenotype. These findings, together with previous observations of defective MMR in pleomorphic STS, indicate that these tumors may be part of the HNPCC-associated tumor spectrum and demonstrate that MMR defects occur in a small subset of STS.

**Key words:** *malignant fibrous histiocytoma, microsatellite instability, mismatch repair, hereditary nonpolyposis colorectal cancer*

### Introduction

Soft tissue sarcomas (STS) are rare with an incidence of 20–25 per million. There are more than 50 histological subtypes of STS, and with increased use of ancillary techniques, such as electron microscopy and immunohistochemical staining, a line of differentiation can be identified in most STS. This development has had particular consequences for malignant fibrous histiocytoma (MFH). MFH was introduced as a specific STS subentity, with fibroblastic appearance but of proposed histiocytic origin, during the 1960s.[1] MFH quickly became the most common STS entity, with the two most common subtypes being the pleomorphic-storiform and the myxoid types.[2] Since its introduction, there has been intense debate among pathologists with a special interest in STS regarding the histogenesis, or line of differentiation shown by MFH and many of these tumors show heterogeneous morphology and multiple, often complex, cytogenetic aberrations.[2,3]

Hereditary nonpolyposis colorectal cancer (HNPCC) is an autosomal dominant inherited disorder that affects 1/1000–2000 individuals.[4,5] HNPCC predisposes for several tumor types, with the greatest life-time risks for colorectal cancer (70–90%), endometrial cancer (40–60%), and increased risks also for cancer of the ovary, ventricle, small intestine, hepatobiliary tract, upper urothelial tract, skin and brain.[6] Clinical criteria, the Amsterdam II criteria, for the classification of HNPCC have been established and include tumor development in at least three individuals in two generations and with one individual diagnosed before age 50. The Amsterdam II criteria consider carcinomas of the colon, rectum, endometrium, small intestine and upper urothelial tract to be closely associated with HNPCC.[7] HNPCC is characterized by a defective mismatch repair (MMR) system, which normally eliminates

Correspondence to: Kajsa Ericson, Department of Oncology, the Jubileum Institution, University Hospital, SE-221 85 Lund, Sweden. Fax: +46–46–147327; E-mail: kajsa.ericson@onk.lu.se

1357-714X print/1369–1643 online © 2004 Taylor & Francis Ltd
DOI: 10.1080/13577140400010856

base–base mismatches and insertion–deletion loops that arise as a consequence of DNA polymerase slippage during DNA replication. HNPCC is caused by MMR gene mutations, with 90% of the mutations affecting the genes *MLH1*, *MSH2*, and *MSH6*.[8,9] Failure to repair DNA mismatches generates small insertion/deletion mutations, which in the tumor tissue appear as altered lengths of small tandem repeats, referred to as microsatellite instability (MSI) and loss of the immunohistochemical expression of the affected MMR protein.[8,10]

A role for defective MMR has been suggested in sarcoma development through observations of occasional sarcomas in HNPCC families and through reports of MMR defects in a subset of STS.[11–15] We therefore applied tissue microarray (TMA) to study the expression of the MMR proteins MLH1, MSH2 and MSH6 in a series of 209 MFH in order to determine a possible contribution of MMR defects in MFH tumorigenesis.

### Material and methods

### *Material*

The Scandinavian Sarcoma Group (SSG) has since 1986 maintained a sarcoma registry, which contains approximately 90% of all diagnosed STS of the extremities and trunk wall. The 209 tumors studied herein have been re-evaluated by the SSG Pathology Review Group (10 Scandinavian pathologists) with access to the clinical history and previous pathology reports. When necessary, electron microscopy and extensive immunohistochemical stainings were performed to exclude a demonstrable lineage of differentiation.[16] The antibody panel included muscle specific actin, smooth muscle actin, desmin, S-100, epithelial membrane antigene, EMA, cytokeratin and several markers specific for melanoma and lymphoma. Malignancy grading was based on a IV-tiered grading system, including the factors cellularity, pleomorphism, nuclear atypia, tumor necrosis, vascular invasion and mitotic activity. The IV-tiered grading system is used by the SSG Pathology Review Group.[25]

Tumor-containing, paraffin-embedded tissue blocks were selected. Core biopsies with a diameter of 0.6 mm were retrieved from the original tumor blocks using a manual analyzer (Beecher Instruments, USA) and were positioned in a recipient paraffin array block. Between three and nine core biopsies were studied from each tumor. The TMA slides were immunostained using antibodies to the MMR proteins MLH1, MSH2 and MSH6 (see below). Tumors that showed inconclusive staining or suspected expression loss in the TMA sections were further immunostained using whole-tissue sections in order to confirm the results. The two tumors that showed loss of the MMR proteins were also subjected to MSI analysis. Ethical approval for the study was obtained from the ethics committee at Lund University.

### *Immunohistochemistry*

Immunohistochemical staining was performed using 4-μm sections of formalin-fixed, paraffin-embedded tissue, which were mounted on DAKO ChemMate Capillary Gap Microscope Slides (DAKO A/S BioTek Solutions, USA) and dried at room temperature overnight followed by 1–2 h at 60°C. The tissue sections were deparaffinized and rehydrated. Antigen retrieval was achieved by microwave-treatment in 1 mM EDTA, pH 9.0, at 900 W for 8 min followed by 15 min at 350 W. The slides were then allowed to cool for at least 20 min in the EDTA solution. Primary antibodies were mouse monoclonal IgG antibodies to MLH1 (clone G168–15, dilution 1:100, PharMingen, San Diego, CA, USA), MSH2 (clone FE-11, dilution 1:100, Oncogene Research Products, Boston, MA, USA) and MSH6 (clone 44, dilution 1:1000, BD Transduction Laboratories). Immunohistochemical staining was performed in an automated immunostainer (TechMate$^{TM}$ 500 Plus, DAKO), according to the manufacturers instructions. DAKO ChemMate Kit peroxidase/3,3′-diaminobenzidine was used for MLH1 and MSH2 and DAKO Envision$^{TM}$/HRP rabbit/mouse for MSH6, with rabbit anti-mouse IgG, dilution 1:400, as a link to amplify between the primary antibody and the Envision step. Diaminobenzidine was used as a chromogen. The sections were counterstained with hematoxylin, dehydrated in ascending concentrations of alcohol to xylene and mounted. A detailed protocol is available from the authors on request. Tumors from HNPCC patients with known germline mutations in *MSH2*, *MLH1* and *MSH6*, respectively, served as controls in each staining round. Loss of expression of the respective MMR protein was defined as absence of nuclear staining in the tumor cells, and normal nuclear staining in lymphocytes and normal epithelial or stromal cells was required serving as internal control (Fig. 1). The expression was classified as present, absent or non-evaluable without grading of the staining intensity.

### *MSI analysis*

DNA was extracted from $3 \times 10$-μm sections of formaldehyde-fixed, paraffin-embedded tissue through incubation of the samples in EDTA–Tris buffer with Proteinase K (at 65°C for at least 2 h, followed by boiling, centrifugation, and removal of the aqueous phase, which was stored at 4°C). The

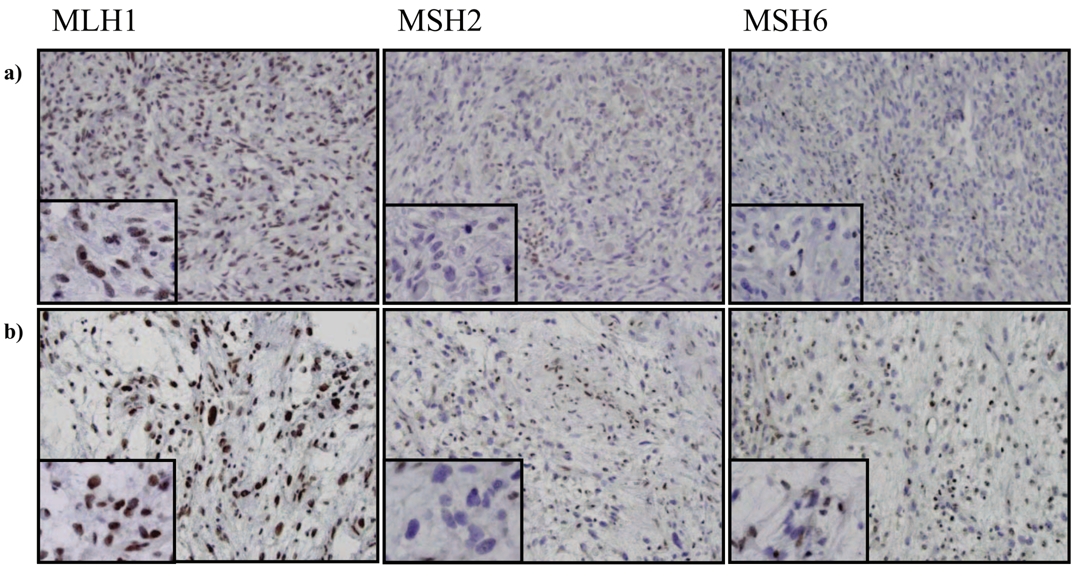

**Fig. 1.** *Loss of immunohistochemical expression of MSH2 and MSH6, with retained expression of MLH1 in a storiform-pleomorphic MFH (a) and a myxoid MFH (b).*

MSI status of the tumors was established using the MSI Multiplex System Prototype Kit (Promega, USA) and with the markers BAT25, BAT26, BAT34C4, D2S123 and D5S346. Data from at least three of the markers were required. The MSI markers used herein have been shown to assess MSI with high accuracy.[17] Details on fluorescent labelling and PCR conditions for the different markers can be obtained from the authors on request. The PCR products were mixed with 12 μl deionized formamide (Hi-Di Formamide, Applied Biosystems) and 0.5 μl ROX™ 500 Size Standard (Applied Biosystems), denatured at 95°C for 2 min, and separated in Performance Optimized Polymer-4 (POP-4™) on an ABI PRISM™ 3100 Genetic Analyzer (Applied Biosystems).

### Results

Of the 209 MFH, 25 tumors showed weak or inconclusive immunostaining. These tumors were further stained using whole-tissue sections. Thereafter, 202 tumors were determined to have normal retained immunostaining for all three antibodies, two tumors showed loss of expression for MSH2 and MSH6, with retained staining for MLH1, and five tumors were non-evaluable due to poor staining quality or lack of staining in the internal control. The two tumors with loss of MSH2 and MSH6 were both high-grade MFH (Fig. 1). The 20-cm storiform-pleomorphic MFH developed in the left thigh of a 96-year-old woman. The tumor was multinodulated and showed a cellular stroma with central necrotic areas and pleomorphic cells with hyperchromatic nuclei and prominent nucleoli. The myxofibrosarcoma was a 7-cm tumor that developed in the right biceps muscle in 77-year-old man. The tumor showed a myxoid stroma with variable cellularity and elongated blood vessels and the fusiform tumor cells showed atypical, enlarged and hyperchromatic nuclei. MSI analysis showed MSI for the markers NR21 and D5S346 in the myxoid MFH, whereas the storiform-pleomorphic MFH did not show MSI for the markers analysed.

### Discussion

We identified loss of MSH2/MSH6 in 2/209 STS and both tumors were high-grade tumor that developed in elderly patients (Fig. 1). The storiform-pleomorphic MFH developed in a woman who is now deceased, but who had not previously been affected by cancer. However, both her father and her brother died from rectal cancer in their seventies, but their tumors were not available for analysis. The myxoid MFH developed in a man who had previously been diagnosed with a prostate cancer, and is now deceased. A family history of cancer was not possible to obtain since he was of non-Scandinavian origin and had no offspring. STS have been associated with several hereditary syndromes that confer an increased risk of cancer, including Werner syndrome due to mutations in the *WRN* gene, Li-Fraumeni syndrome due to germline *TP53* mutations, and other rare syndromes such as Rothmund–Thomson syndrome and Bloom syndrome.[11,18–20]

HNPCC is estimated to occur at a population frequency of 1/1000–2000 and predisposes to a wide spectrum of tumors, including colorectal cancer, endometrial cancer, cancer of the ovary, ventricle, small intestine, hepatobiliary tract, upper urinary tract, skin, and brain.[6] Sarcomas are generally not considered to occur in the context of HNPCC, but have been reported to occur in 1–2% of HNPCC patients.[21,22] Sarcoma development in HNPCC patients have been reported in four case reports, and included three MFH and a pleomorphic rhabdomyosarcoma.[11,13,14,21] The two tumors that were genetically investigated (one MFH and the rhabdomyosarcoma) showed MSI and immunohistochemical loss of MSH2 expression. Our findings, together with the previous reports of MMR-defective sarcomas with loss of MSH2, demonstrate that sarcomas may represent a rare tumor type in HNPCC families. So far, the sarcomas have specifically been associated with inactivation of MSH2/MSH6, and a higher risk of extraintestinal tumors have indeed been described in HNPCC families with mutations affecting the MSH2 gene.[23] Since MSH2 and MSH6 functionally interact, a simultaneous loss of both proteins is expected.[24] Loss of MSH2 expression has so far exclusively been associated with germline mutations in colorectal cancer. However, somatic mutations could occur in other tumor types.

The possible role for defective MMR in the development of non-HNPCC-associated STS is unclear. Our study does not indicate that defective MMR should be a major tumorigenic mechanism in these tumors. However, Suwa *et al.* found MSI in three of 39 STS (a liposarcoma, a synovial sarcoma and a leiomyosarcoma),[15] and Saito *et al.* reported a MSI low phenotype and loss of MLH1/MSH2 expression in five of 11 alveolar soft part sarcomas (ASPS).[12] Perhaps different genetic mechanisms apply, with the MSI-high tumors representing only the small fraction of the STS that are associated with HNPCC, whereas a larger subset of STS may display instability at a single loci and thus be of the MSI-low phenotype.

In summary, a subset of STS develop through defective MMR, and MFH may occur as a rare tumor within the HNPCC tumor spectrum. This implicates that HNPCC testing may be performed in family members who have developed STS and suggests that genetic counseling should be offered patients with STS who reveal a family history of HNPCC-associated tumors.

### Acknowledgements

We would like to acknowledge the pathologists in the Scandinavian Sarcoma Group review board for performing the histopathological review of the tumors included. Cecilia Östermalm is acknowledged for technical assistance, and Jeff Becher for providing the MSI Multiplex System Prototype Kit. Financial support was granted from the Swedish Cancer Society, the Nilsson Cancer Fund, the Kamprad Fund and the Lund University Hospital Cancer Funds.

### References

1. Ozzello L, Stout AP, Murray MR. Cultural characteristics of malignant histiocytomas and fibrous xanthomas. *Cancer* 1963; 16: 331–44.
2. Fletcher CUK, Mertens F. *Pathology and Genetics, Tumours of Soft Tissue and Bone*. Lyon, France: IARC Press, 2002.
3. Fletcher CD, Fletcher JA, Cin PD, Ladanyi M, Woodruff JM. Diagnostic gold standard for soft tissue tumours: morphology or molecular genetics? *Histopathology* 2001; 39(1): 100–3.
4. Samowitz WS, Curtin K, Lin HH, Robertson MA, Schaffer D, Nichols M, *et al.* The colon cancer burden of genetically defined hereditary nonpolyposis colon cancer. *Gastroenterology* 2001; 121(4): 830–8.
5. Aaltonen LA, Salovaara R, Kristo P, Canzian F, Hemminki A, Peltomaki P, *et al.* Incidence of hereditary nonpolyposis colorectal cancer and the feasibility of molecular screening for the disease. *New Engl J Med* 1998; 338(21): 1481–7.
6. Aarnio M, Sankila R, Pukkala E, Salovaara R, Aaltonen LA, de la Chapelle A, *et al.* Cancer risk in mutation carriers of DNA-mismatch-repair genes. *Int J Cancer* 1999; 81(2): 214–8.
7. Vasen HF, Watson P, Mecklin JP, Lynch HT. New clinical criteria for hereditary nonpolyposis colorectal cancer (HNPCC, Lynch syndrome) proposed by the International Collaborative group on HNPCC. *Gastroenterology* 1999; 116(6): 1453–6.
8. Peltomaki P. Deficient DNA mismatch repair: a common etiologic factor for colon cancer. *Hum Mol Genet* 2001; 10(7): 735–40.
9. Mitchell RJ, Farrington SM, Dunlop MG, Campbell H. Mismatch repair genes hMLH1 and hMSH2 and colorectal cancer: a HuGE review. *Am J Epidemiol* 2002; 156(10): 885–902.
10. Aaltonen LA, Sankila R, Mecklin JP, Jarvinen H, Pukkala E, Peltomaki P, *et al.* A novel approach to estimate the proportion of hereditary nonpolyposis colorectal cancer of total colorectal cancer burden. *Cancer Detect Prev* 1994; 18(1): 57–63.
11. Lynch HT, Deters CA, Hogg D, Lynch JF, Kinarsky Y, Gatalica Z. Familial sarcoma: challenging pedigrees. *Cancer* 2003; 98(9): 1947–57.
12. Saito T, Oda Y, Kawaguchi K, Takahira T, Yamamoto H, Sakamoto A, *et al.* Possible association between tumor-suppressor gene mutations and hMSH2/hMLH1 inactivation in alveolar soft part sarcoma. *Hum Pathol* 2003; 34(9): 841–9.
13. Sijmons R, Hofstra R, Hollema H, Mensink R, van der Hout A, Hoekstra H, *et al.* Inclusion of malignant fibrous histiocytoma in the tumour spectrum associated with hereditary non-polyposis colorectal cancer. *Genes Chromosomes Cancer* 2000; 29(4): 353–5.
14. Den Bakker MA, Seynaeve C, Kliffen M, Dinjens WN. Microsatellite instability in a pleomorphic rhabdomyosarcoma in a patient with hereditary non-polyposis colorectal cancer. *Histopathology* 2003; 43(3): 297–9.
15. Suwa K, Ohmori M, Miki H. Microsatellite alterations in various sarcomas in Japanese patients. *J Orthop Sci* 1999; 4(3): 223–30.
16. Meis-Kindblom JM, Bjerkehage B, Bohling T, Domanski H, Halvorsen TB, Larsson O, *et al.* Morphologic review of 1000 soft tissue sarcomas from the Scandinavian Sarcoma Group (SSG) Register. The peer-review committee experience. *Acta Orthop Scand Suppl* 1999; 285: 18–26.

17. Boland CR, Thibodeau SN, Hamilton SR, Sidransky D, Eshleman JR, Burt RW, *et al.* A National Cancer Institute Workshop on Microsatellite Instability for cancer detection and familial predisposition: development of international criteria for the determination of microsatellite instability in colorectal cancer. *Cancer Res* 1998; 58(22): 5248–57.
18. Vennos EM, Collins M, James WD. Rothmund-Thomson syndrome: review of the world literature. *J Am Acad Dermatol* 1992; 27(5 Pt 1): 750–62.
19. Goto M, Miller RW, Ishikawa Y, Sugano H. Excess of rare cancers in Werner syndrome (adult progeria). *Cancer Epidemiol Biomarkers Prev* 1996; 5(4): 239–46.
20. Li FP, Correa P, Fraumeni JF Jr. Testing for germ line p53 mutations in cancer families. *Cancer Epidemiol Biomarkers Prev* 1991; 1(1): 91–4.
21. Mecklin JP, Jarvinen HJ. Tumor spectrum in cancer family syndrome (hereditary nonpolyposis-colorectal cancer). *Cancer* 1991; 68(5): 1109–12.
22. Aarnio M, Mecklin JP, Aaltonen LA, Nystrom-Lahti M, Jarvinen HJ. Life-time risk of different cancers in hereditary non-polyposis colorectal cancer (HNPCC) syndrome. *Int J Cancer* 1995; 64(6): 430–3.
23. Vasen HF, Stormorken A, Menko FH, Nagengast FM, Kleibeuker JH, Griffioen G, *et al.* MSH2 mutation carriers are at higher risk of cancer than MLH1 mutation carriers: a study of hereditary nonpolyposis colorectal cancer families. *J Clin Oncol* 2001; 19(20): 4074–80.
24. Acharya S, Wilson T, Gradia S, Kane MF, Guerrette S, Marsischky GT, *et al.* hMSH2 forms specific mispair-binding complexes with hMSH3 and hMSH6. *Proc Natl Acad Sci USA* 1996; 93(24): 13629–34.
25. Markhede G, Angerwall L, Sterner B. A multivariate analysis of the prognosis after surgical treatment of malignant soft-tissue tumours. *Cancer* 1982; 49: 1721–33.

## References

[PDF_00358] Aaltonen L. A., Salovaara R., Kristo P., Canzian F., Hemminki A., Peltomäki P., Chadwick R. B., Käriäinen H., Eskelinen M., Järvinen H. (1998). Incidence of hereditary nonpolyposis colorectal cancer and the feasibility of molecular screening for the disease.. N Engl J Med.

[PDF_00379] Aaltonen L. A., Sankila R., Mecklin J. P., Järvinen H., Pukkala E., Peltomäki P., de la Chapelle A. (1994). A novel approach to estimate the proportion of hereditary nonpolyposis colorectal cancer of total colorectal cancer burden.. Cancer Detect Prev.

[PDF_00434] Aarnio M., Mecklin J. P., Aaltonen L. A., Nyström-Lahti M., Järvinen H. J. (1995). Life-time risk of different cancers in hereditary non-polyposis colorectal cancer (HNPCC) syndrome.. Int J Cancer.

[PDF_00363] Aarnio M., Sankila R., Pukkala E., Salovaara R., Aaltonen L. A., de la Chapelle A., Peltomäki P., Mecklin J. P., Järvinen H. J. (1999). Cancer risk in mutation carriers of DNA-mismatch-repair genes.. Int J Cancer.

[PDF_00445] Acharya S., Wilson T., Gradia S., Kane M. F., Guerrette S., Marsischky G. T., Kolodner R., Fishel R. (1996). hMSH2 forms specific mispair-binding complexes with hMSH3 and hMSH6.. Proc Natl Acad Sci U S A.

[PDF_00413] Boland C. R., Thibodeau S. N., Hamilton S. R., Sidransky D., Eshleman J. R., Burt R. W., Meltzer S. J., Rodriguez-Bigas M. A., Fodde R., Ranzani G. N. (1998). A National Cancer Institute Workshop on Microsatellite Instability for cancer detection and familial predisposition: development of international criteria for the determination of microsatellite instability in colorectal cancer.. Cancer Res.

[PDF_00350] Fletcher C. D., Fletcher J. A., Dal Cin P., Ladanyi M., Woodruff J. M. (2001). Diagnostic gold standard for soft tissue tumours: morphology or molecular genetics?. Histopathology.

[PDF_00424] Goto M., Miller R. W., Ishikawa Y., Sugano H. (1996). Excess of rare cancers in Werner syndrome (adult progeria).. Cancer Epidemiol Biomarkers Prev.

[PDF_00428] Li F. P., Correa P., Fraumeni J. F. (1991). Testing for germ line p53 mutations in cancer families.. Cancer Epidemiol Biomarkers Prev.

[PDF_00384] Lynch Henry T., Deters Carolyn A., Hogg David, Lynch Jane F., Kinarsky Yulia, Gatalica Zoran (2003). Familial sarcoma: challenging pedigrees.. Cancer.

[PDF_00449] Markhede G., Angervall L., Stener B. (1982). A multivariate analysis of the prognosis after surgical treatment of malignant soft-tissue tumors.. Cancer.

[PDF_00431] Mecklin J. P., Järvinen H. J. (1991). Tumor spectrum in cancer family syndrome (hereditary nonpolyposis colorectal cancer).. Cancer.

[PDF_00406] Meis-Kindblom J. M., Bjerkehage B., Böhling T., Domanski H., Halvorsen T. B., Larsson O., Lilleng P., Myhre-Jensen O., Stenwig E., Virolainen M. (1999). Morphologic review of 1000 soft tissue sarcomas from the Scandinavian Sarcoma Group (SSG) Register. The peer-review committee experience.. Acta Orthop Scand Suppl.

[PDF_00375] Mitchell R. J., Farrington S. M., Dunlop M. G., Campbell H. (2002). Mismatch repair genes hMLH1 and hMSH2 and colorectal cancer: a HuGE review.. Am J Epidemiol.

[PDF_00344] OZZELLO L., STOUT A. P., MURRAY M. R. (1963). Cultural characteristics of malignant histiocytomas and fibrous xanthomas.. Cancer.

[PDF_00372] Peltomäki P. (2001). Deficient DNA mismatch repair: a common etiologic factor for colon cancer.. Hum Mol Genet.

[PDF_00387] Saito Tsuyoshi, Oda Yoshinao, Kawaguchi Ken-Ichi, Takahira Tomonari, Yamamoto Hidetaka, Sakamoto Akio, Tamiya Sadafumi, Iwamoto Yukihide, Tsuneyoshi Masazumi (2003). Possible association between tumor-suppressor gene mutations and hMSH2/hMLH1 inactivation in alveolar soft part sarcoma.. Hum Pathol.

[PDF_00354] Samowitz W. S., Curtin K., Lin H. H., Robertson M. A., Schaffer D., Nichols M., Gruenthal K., Leppert M. F., Slattery M. L. (2001). The colon cancer burden of genetically defined hereditary nonpolyposis colon cancer.. Gastroenterology.

[PDF_00392] Sijmons R., Hofstra R., Hollema H., Mensink R., van der Hout A., Hoekstra H., Kleibeuker J., Molenaar W., Wijnen J., Fodde R. (2000). Inclusion of malignant fibrous histiocytoma in the tumour spectrum associated with hereditary non-polyposis colorectal cancer.. Genes Chromosomes Cancer.

[PDF_00403] Suwa K., Ohmori M., Miki H. (1999). Microsatellite alterations in various sarcomas in Japanese patients.. J Orthop Sci.

[PDF_00439] Vasen H. F., Stormorken A., Menko F. H., Nagengast F. M., Kleibeuker J. H., Griffioen G., Taal B. G., Moller P., Wijnen J. T. (2001). MSH2 mutation carriers are at higher risk of cancer than MLH1 mutation carriers: a study of hereditary nonpolyposis colorectal cancer families.. J Clin Oncol.

[PDF_00367] Vasen H. F., Watson P., Mecklin J. P., Lynch H. T. (1999). New clinical criteria for hereditary nonpolyposis colorectal cancer (HNPCC, Lynch syndrome) proposed by the International Collaborative group on HNPCC.. Gastroenterology.

[PDF_00421] Vennos E. M., Collins M., James W. D. (1992). Rothmund-Thomson syndrome: review of the world literature.. J Am Acad Dermatol.

[PDF_00398] den Bakker M. A., Seynaeve C., Kliffen M., Dinjens W. N. M. (2003). Microsatellite instability in a pleomorphic rhabdomyosarcoma in a patient with hereditary non-polyposis colorectal cancer.. Histopathology.

